# Supplementary figures and images for: A Personalized Approach to Percutaneous Coronary Interventions in the Left Main Coronary Artery—Is the Female Gender Associated with Worse Outcomes?
Source: J Pers Med. 2021 Jun 20;11(6):581. doi: 10.3390/jpm11060581 (PMC8235057; doi:10.3390/jpm11060581)

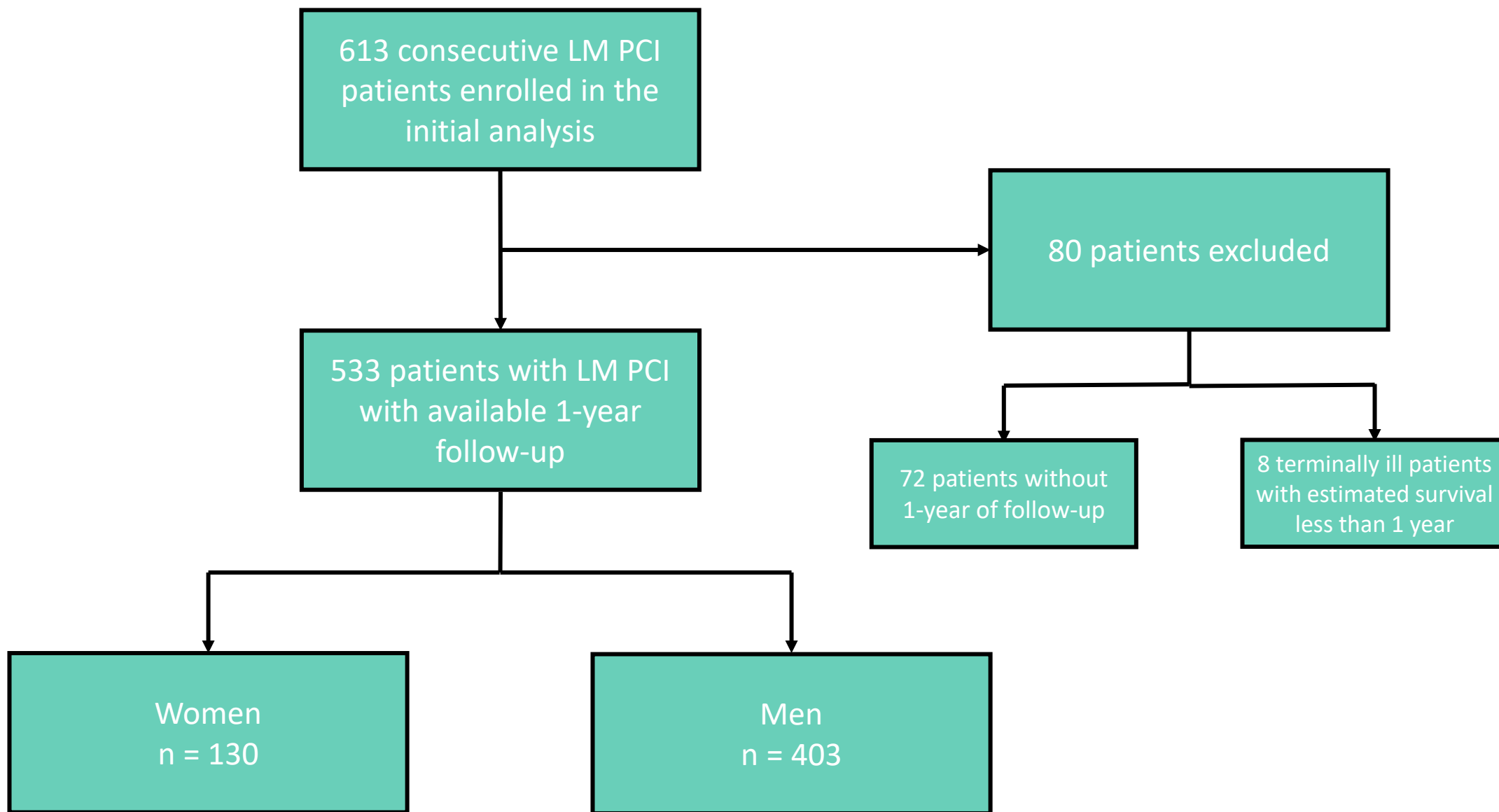

Supplement: Supplementary file 1 [file jpm-11-00581-s001.zip › jpm-1247459-supplementary.pdf]
